# Telomeres and replicative cellular aging of the human placenta and chorioamniotic membranes

**DOI:** 10.1038/s41598-021-84728-2

**Published:** 2021-03-04

**Authors:** Tsung-Po Lai, Mark Simpson, Krunal Patel, Simon Verhulst, Jungsik Noh, Natalie Roche, Debra Heller, George Guirguis, Jerry W. Shay, Utz Herbig, Abraham Aviv

**Affiliations:** 1 Center of Human Development and Aging (F-464 MSB), New Jersey Medical School, Rutgers, The State University of New Jersey, 185 South Orange Ave, Newark, NJ 07103 USA; 2 Department of Microbiology and Molecular Genetics, New Jersey Medical School, Rutgers, The State University of New Jersey, Newark, NJ USA; 3 Department of Obstetrics, Gynecology and Women’s Health, New Jersey Medical School, Rutgers, The State University of New Jersey, Newark, NJ USA; 4 Groningen Institute for Evolutionary Life Sciences, University of Groningen, Groningen, The Netherlands; 5 Department of Bioinformatics, University of Texas Southwestern Medical Center, Dallas, TX USA; 6 Department of Pathology and Laboratory Medicine, New Jersey Medical School, Rutgers The State University of New Jersey, Newark, NJ USA; 7 Department of Cell Biology, University of Texas Southwestern Medical Center, Dallas, TX USA

**Keywords:** Ageing, Biomarkers

## Abstract

Recent hypotheses propose that the human placenta and chorioamniotic membranes (CAMs) experience telomere length (TL)-mediated senescence. These hypotheses are based on mean TL (mTL) measurements, but replicative senescence is triggered by short and dysfunctional telomeres, not mTL. We measured short telomeres by a vanguard method, the Telomere shortest length assay, and telomere-dysfunction-induced DNA damage foci (TIF) in placentas and CAMs between 18-week gestation and at full-term. Both the placenta and CAMs showed a buildup of short telomeres and TIFs, but not shortening of mTL from 18-weeks to full-term. In the placenta, TIFs correlated with short telomeres but not mTL. CAMs of preterm birth pregnancies with intra-amniotic infection showed shorter mTL and increased proportions of short telomeres. We conclude that the placenta and probably the CAMs undergo TL-mediated replicative aging. Further research is warranted whether TL-mediated replicative aging plays a role in all preterm births.

## Introduction

Recent hypotheses posit that during human gestation, the placenta and the chorioamniotic membranes (CAMs), i.e., the chorionic membrane (CM) and amniotic membrane (AM), experience ‘replicative aging’ that is driven by the shortening of telomeres^1,2^. This aging presumably proceeds through an accelerated process, analogous in many respects to the protracted aging of replicating somatic tissues during extra-uterine life. Telomere length (TL)-mediated replicative aging of the placenta and CAMs, these hypotheses further propose, might be a common mechanistic pathway that contributes to the timing of parturition. The following question, however, illustrates the knowledge gap in understanding of TL-mediated replicative aging during human gestation: How can the placenta be an aging organ at the end of gestation when its mTL is longer than mTL of leukocytes in the umbilical cord blood (UCB) of the newborn^3,4^?

We propose that these ‘replicative aging’ hypotheses of the placenta and CAMs require revisions based on the following premise: The shortest telomeres^5,6^, expressed in dysfunctional telomeres^7–10^ and their buildup, rather than mTL, are the indicators of telomere-mediated aging of the placenta and CAMs, as their biological utility draws to an end. We proceeded as follows to test this idea: First, we obtained samples at the end of normal (full term) pregnancies from the placenta, the CM and AM, UCB and maternal blood from mother-newborn pairs. In these samples and all other samples collected in this study, we (a) measured the mean length of the terminal restriction fragments (TRFs), generated by Southern blotting (SB)^11^, and (b) measured and tallied the TRFs by the newly-developed Telomere Shortest Length Assay (TeSLA)^12^. Moreover, we quantified telomere dysfunction-induced DNA damage foci (TIF)^10^ in all placental and CAM samples. Second, we measured TL parameters by SB and TeSLA, and quantified TIF in placenta and CAMs samples obtained after elective abortion at mean gestational age of 18 weeks. Results were then compared with findings at full term. Third, in a pilot study, we measured TL parameters by SB and TeSLA in placenta and CAMs at birth in (a) uneventful full-term pregnancies, (b) pregnancies complicated by preterm birth (PTB), (c) PTB with intra-amniotic inflammation/infection (PTBI), and (d) pregnancies complicated by small-for-gestational age (SGA) newborns.

## Results

### Characteristics of TL parameters measured by SB and TeSLA, and telomere dysfunction measured by TIF

The principle measurements employed in this work (SB, TeSLA and TIF) provide different but vital information about the potential role of TL-mediated replicative aging in the biology of the human placenta and CAMs. Although the SB method is the ‘gold standard’ of TL measurements against which the validity of all other techniques have been judged^11^, it was originally designed to measure mTL, i.e., SBmTL, which reflects the mean length of the 92 telomeres of the q and p arms of the 23 human chromosome pairs. When used in population-based telomere research and in this study (Fig. S1a), SB typically generates a weak TRF signal below 3 kb; the analysis is applied, therefore, to a range of 3–20 kb^11^. TeSLA, in contrast, generates a signal of TRFs for single telomeres that extends below 3 kb (Fig. S1b)^12^. Although the two methodologies of TL measurements inter-relate, they do not produce identical information, because the TeSmTL includes telomeres shorter than 3 kb (Fig. S2).

In the final analysis, the buildup of short telomeres is expressed in telomere dysfunction, which is captured by TIF^7–10^. We found it essential, therefore, to examine the relation in the placenta and CAMs between TL parameters and TIF. The key for this analysis is the simultaneous signals of DNA damage response (DDR), as detected using antibodies against p53 binding protein 1 (53BP1), and telomeres labeled with a Cy3-conjugated peptide nucleic acid (PNA) complementary to telomeric repeats (illustrated in Fig. S3).

### TL parameters and TIF at the end of normal, full-term pregnancies

**Figure 1:**
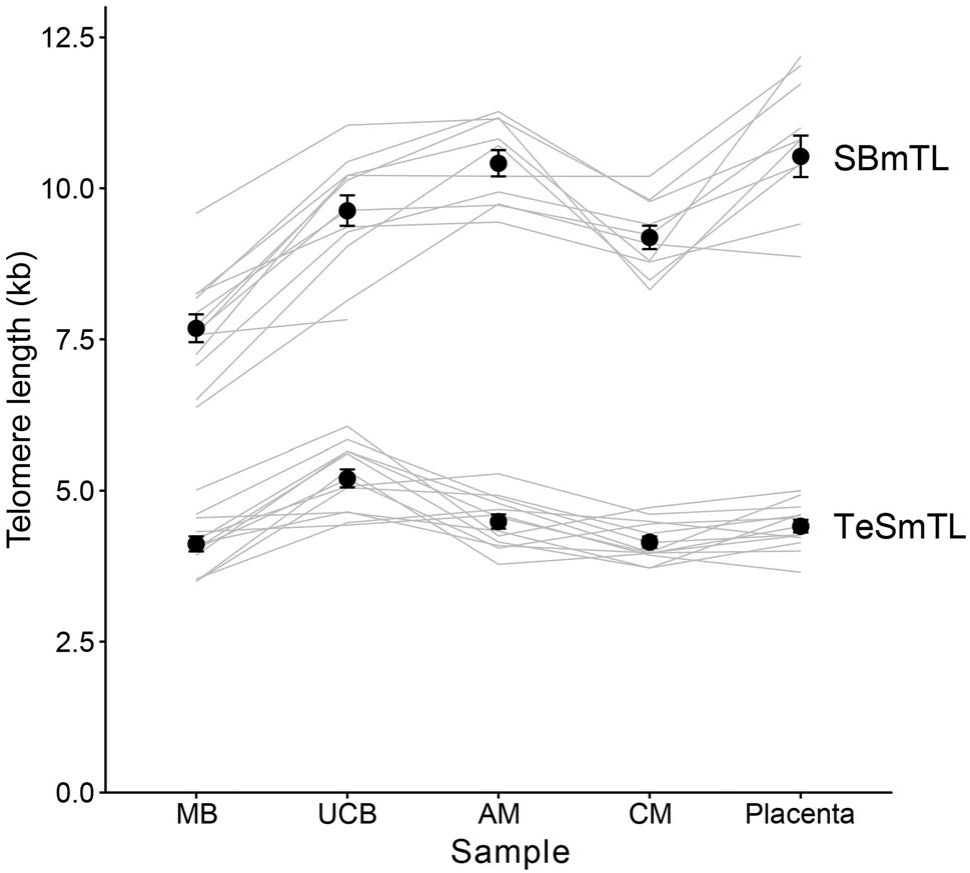
Mean telomere length (kb) in different tissues at full-term, measured using Southern blotting (SBmTL) and TeSLA (TeSmTL). Grey lines connect measurements on different tissues of the same mother–offspring pair (n = 13), markers show average ± SE. samples: maternal blood (MB), umbilical cord blood (UCB), amniotic membrane (AM), chorionic membrane (CM), Placenta.

SBmTL varied significantly between tissues within subjects (mixed model, including subject as random effect: tissue: F_4,42.18_ = 39.59, *P* < 0.0001; Fig. 1). Moreover, there were substantial inter-individual differences in SBmTL across tissues (ICC individual identity: 0.51, SE = 0.16). Pairwise comparisons (Tukey method) revealed that placental SBmTL (10.53 ± 0.34 kb; mean ± SE) was longer than SBmTL of UCB (9.63 ± 0.25 kb; *t* = 3.58, *P* = 0.007). SBmTL in maternal blood (7.69 ± 0.23 kb) was shorter than SBmTL in all other samples; all *t* > 5.1, *P* < 0.0002).

SBmTL differed between CAMs, being longer in AM than the CM (Fig. 1; *t* = 4.29, *P* = 0.0009). It was shorter in the CM than in the placenta (*t* = 5.25, *P* < 0.0001), and did not differ from SBmTL in UCB (*t* = 1.97, *P* = 0.30). In contrast, SBmTL in the AM did not differ from SBmTL in placental samples (*t* = 0.77, *P* = 0.94), or in UCB (*t* = 2.52, *P* = 0.11). An overview of all pair-wise comparisons is provided in Table S1A.

TeSmTL at full term also varied significantly between tissues (mixed model, including subject as random effect: tissue: F_4,48.0_ = 17.07, *P* < 0.0001; Fig. 1). This was due to TeSmTL being longer in UCB compared to all other tissues (all *t* > 4.7, *P* < 0.003), while pair-wise comparisons revealed no significant differences in TeSmTL between the other tissues (all *t* > 2.47, *P* > 0.11). A comparison between tissues of the proportion of telomeres shorter than 3.0 kb yielded the same result, with significant variation between tissues (F_4,48_ = 19.98, *P* < 0.0001) which was due to fewer short telomeres in UCB compared with all other tissues (all *t* > 5.1, *P* < 0.0002), and no significant differences between the other tissues (all *t* < 2.77). An overview of all pair-wise comparisons is provided in Table S1B. There were modest inter-individual differences in TeSmTL across tissues (ICC individual identity: 0.21, SE = 0.13).

Placental findings above are for samples obtained from the central placenta. Previous work found no SBmTL difference between the central and peripheral placenta ^3^, but we found a trend for SBmTL to be longer in central placenta samples compared to peripheral samples (Fig. S4; paired-t-test; difference ± SE: 0.47 ± 0.22 kb, *t*_12_ = 2.09, *P* = 0.059). However, there was no such pattern for the TeSmTL (paired-t-test; difference ± SE: 0.08 ± 0.093 kb, *t*_12_ = − 0.86, *P* = 0.40), consistent with previous single TL analysis of the p arms of the X and Y chromosomes ^13^.

**Figure 2:**
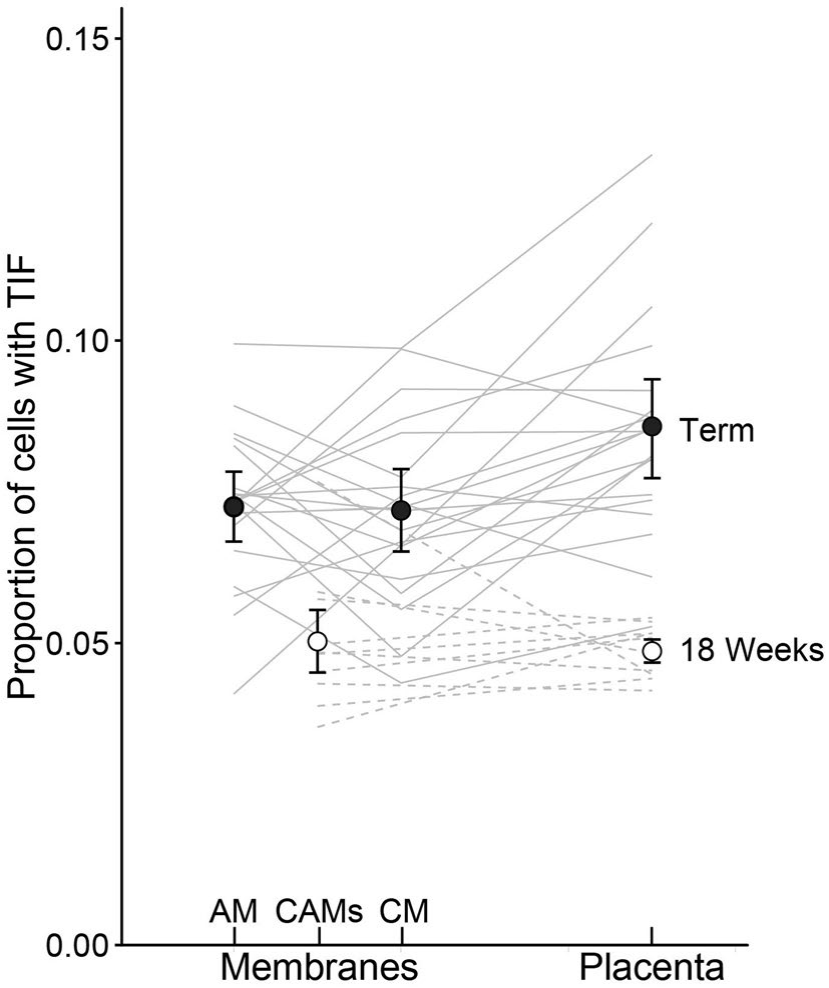
Proportion of cells with telomere dysfunction induced foci (TIF) at 18-weeks of gestation (n = 10) and at term (n = 18), in the placenta and the chorioamniotic membranes (CAMs). Note that at 18 weeks the CAMs could not be separated and hence the measurements are for the chorionic membrane (CM) and amniotic membrane (AM) combined, i.e., CAMs. Lines connect measurements in different tissues from the same donor, and dots show means ± SE.

At full term, the proportion of cells with TIF, measured in the placenta and the CAMs, differed significantly between tissues (Fig. 2; F_2,38_ = 7.48, *P* < 0.002). This was due to the proportion of cells with TIF being higher in placenta compared to both the CM and AM (*t* > 3.2, *P* < 0.007), while there was no difference in the proportion of cells with TIF between the CM and AM (*t* = 0.21, *P* = 0.98).

### TL parameters and TIF at 18-week gestation

We examined the CAMs as a single tissue at 18-weeks of gestation (gestational age 18.2 ± 2.57; mean ± SD; n = 10) because of technical difficulties of separating the CM from the AM at early stages of pregnancy. There were substantial inter-individual differences in SBmTL across tissues (ICC individual identity ± SE: 0.45 ± 0.23). There was a trend for SBmTL_,_ to be longer in placenta compared to the CAMs within subjects (paired-*t*-test, difference ± SE: 0.75 ± 0.34 kb, *t*_9_ = 2.91, *P* = 0.056), but there were no differences in TeSmTL across tissues (ICC = 0.0 ± 0.19). Consistent with the absence of a difference in TeSmTL, there was also no difference in the proportion of telomeres shorter than 3.0 kb or in TIF between membranes and placenta at 18 weeks of gestation (proportion < 3.0 kb: *t*_9_ = 0.89, *P* = 0.40; TIF: *t*_9_ = 0.32, *P* = 0.76).

### Changes in TL parameters and TIF during gestation

**Figure 3:**
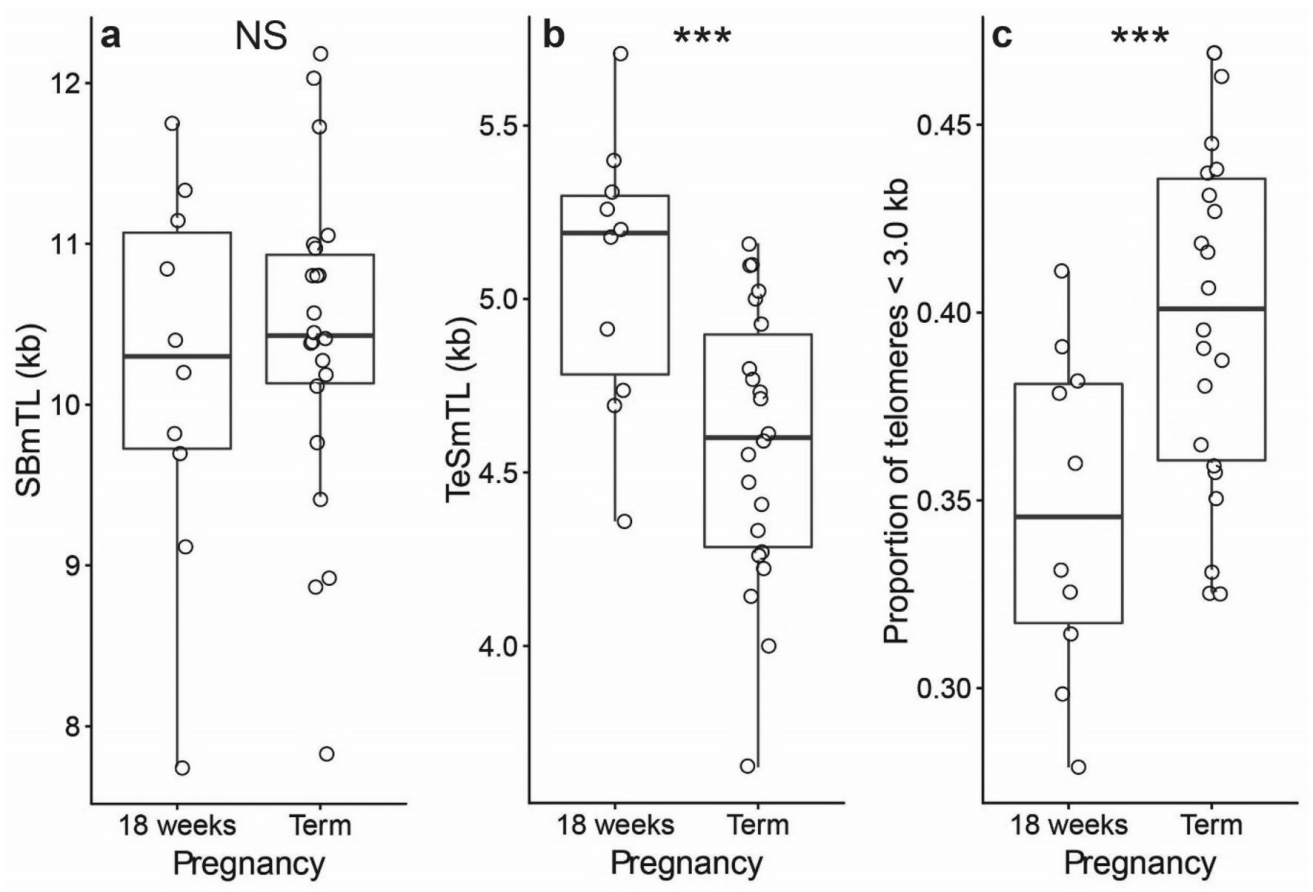
Telomere length in the placenta at 18-weeks of gestation (n = 10) and at full term (n = 22). Southern blot mean telomere length (SBmTL) (**a**); TeSLA mean telomere length (TeSmTL) (**b**); proportion of telomeres shorter than 3.0 kb (TeSLA measurements) (**c**). NS, not significant; ***P < 0.001.

We compared SBmTL, TeSmTL and TIF in a set of placentas and CAMs between 18-weeks of gestation (n = 10) and full term. SBmTL of placenta samples did not differ significantly between gestational stages (Fig. 3a; difference ± SE: + 0.33 ± 0.51 kb at term; *t*_21_ = 0.64, *P* = 0.53). However, TeSmTL was significantly shorter at full term (Fig. 3b; − 0.66 ± 0.16 kb; *t*_21_ = 4.09, *P* = 0.0005), which was at least partially due to a higher proportion of telomeres shorter than 3.0 kb at full term (Fig. 3c; *t* = 3.83, *P* < 0.001).

**Figure 4:**
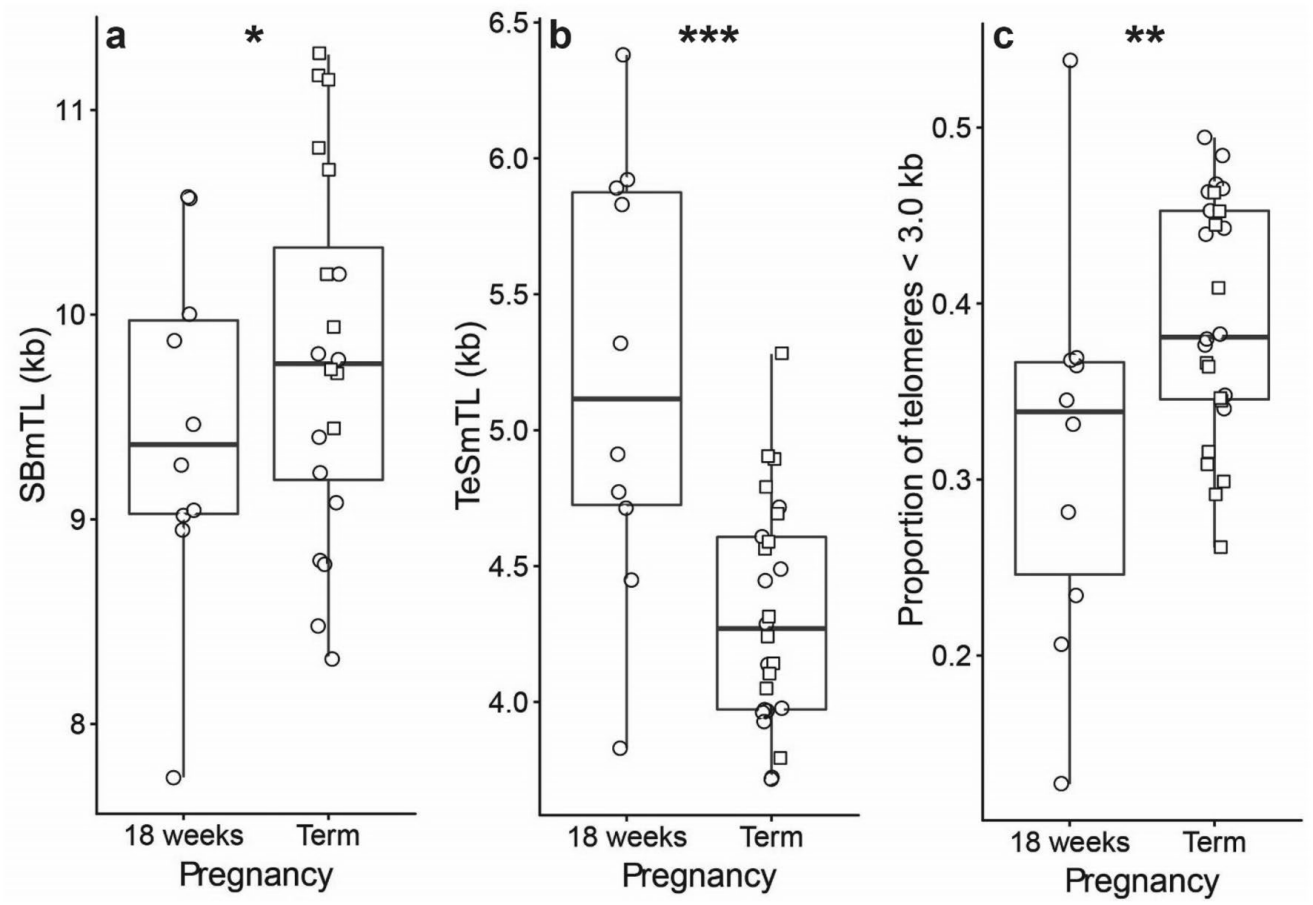
Telomere length in the chorioamniotic membranes (CAMs) at 18-weeks of gestation (n = 10) and at full term (n = 13). Southern blot mean telomere length (SBmTL) (**a**); TeSLA mean telomere length (TeSmTL) (**b**); proportion of telomeres shorter than 3.0 kb (TeSLA measurements) (**c**). Note that at 18 weeks the measurements are for the chorionic membrane (CM) and amniotic membrane (AM) combined, i.e., CAMs, since the two could not be separated. At term the two membranes could be separated (AM, squares; CM, circles), but boxplot is for the membranes pooled. *P < 0.05; **P < 0.01; ***P < 0.001.

SBmTL in the AM was significantly longer at full term than in the CAMs at 18 weeks (Fig. 4a; + 0.97 ± 0.35 kb; *t*_18_ = 2.78, *P* = 0.012), while in the CM at full term, SBmTL did not differ significantly from that in the CAMs at 18 weeks (− 0.26 ± 0.33 kb; *t*_18_ = 0.79, *P* = 0.44). In contrast, TeSmTLs in both the AM and the CM were shorter at full term compared to the CAMs at gestation age 18 weeks (Fig. 4b; AM: − 0.71 ± 0.26 kb, *t*_21_ = 2.75, *P* = 0.012; CM: − 1.05 ± 0.24 kb, *t*_21_ = 4.31, *P* = 0.0003). Consistent with this result, the proportion of telomeres shorter than 3.0 kb in CM was higher at full term than in the CAMs at gestation age 18 weeks (Fig. 4c; *t*_21_ = 3.09, *P* = 0.006), but this comparison was not significant for the AM (*t*_21_ = 1.21, *P* = 0.24).

The proportion of cells in placenta showing TIF was higher at full term than at 18 weeks (Fig. 2; *t*_28_ = 6.92, *P* < 0.001). The proportion of cells in the CAMs showing TIF was also higher at full term than at 18 weeks (AM at term versus CAMs at 18 weeks: *t*_28_ = 4.73, *P* < 0.0001; the same comparison for CM: *t*_28_ = 4.11, *P* < 0.0001). The effect of pregnancy stage on TIF was stronger in placenta compared to the CAMs (interaction tissue * pregnancy stage: F_1,74_ = 5.87, *P* = 0.023).

**Figure 5:**
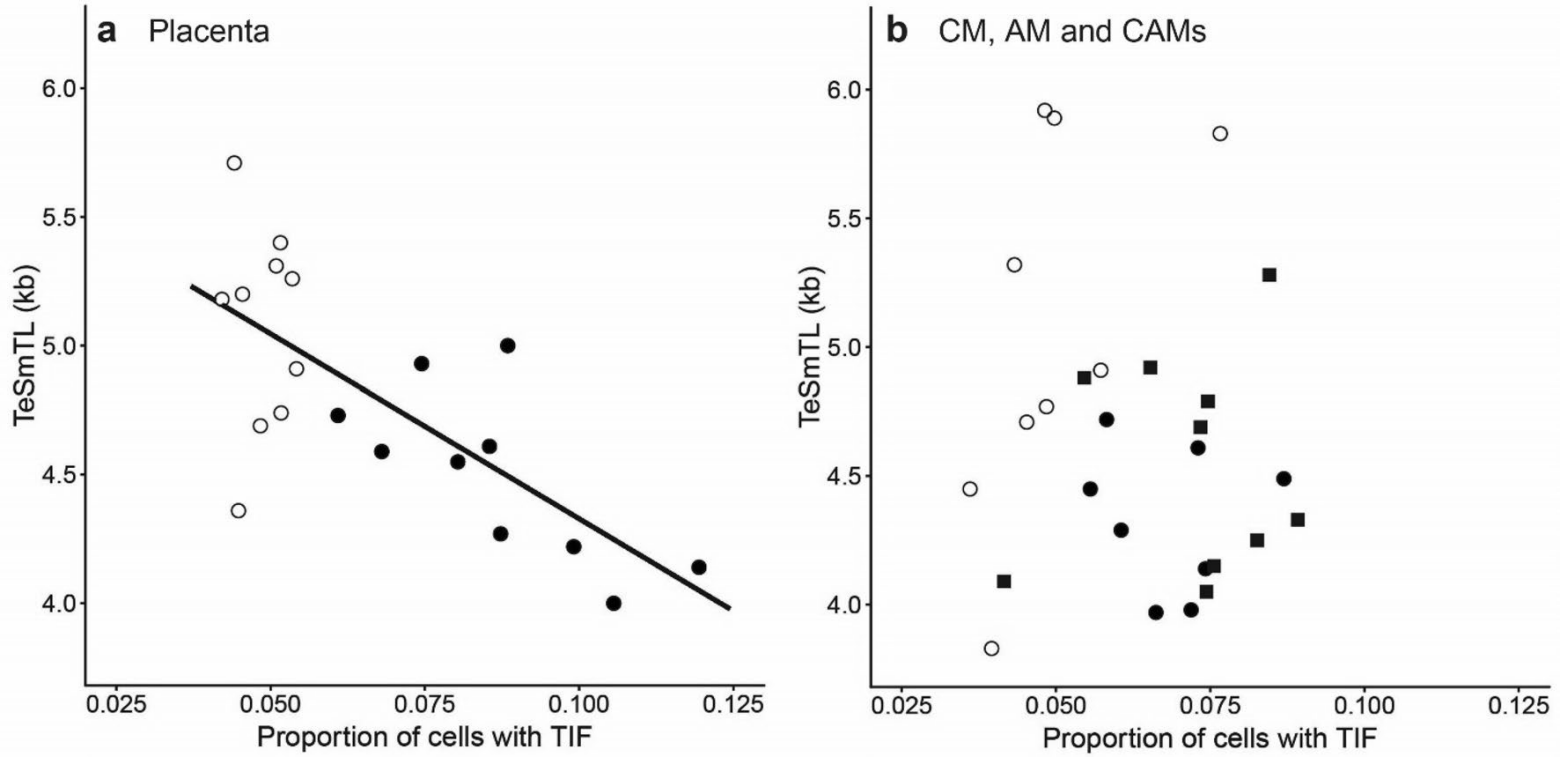
Correlation between telomere dysfunction induced foci (TIF) and TeSLA mTL (TeSmTL). Placenta (**a**); chorioamniotic membranes (CAMs) (**b**). For both panels (**a**) and (**b**) open data symbols: samples at 18-week gestation, filled data symbols: samples at term. For panel (**b**), chorionic membrane (CM, black circles), amniotic membrane (AM, black squares). For panel (**a**) correlation (R^2^ = 0.52, n = 20, *P* < 0.001) between TeSmTL and proportion of TIF, and *P* = 0.41 (n = 27) for panel (**b**).

TeSmTL was shorter in placenta samples with a higher proportion of cells with TIF (Fig. 5a; linear regression *t*_18_ = 4.40, *P* = 0.0003) and this association was independent of pregnancy stage (*P* = 0.8 when added to a regression model containing TIF only). The same result emerges when TeSmTL is replaced with the proportion of telomeres shorter than 3.0 kb (linear regression *t*_18_ = 3.36, *P* = 0.003). In CAMs, there was no significant association of TeSmTL with the proportion of cells with a TIF when results of the AM and the CM were pooled (Fig. 5b; t_28_ = 0.84, *P* = 0.41), and this did not change selecting either AM or CM at full term (*P* > 0.33), or when replacing TeSmTL with the proportion of telomeres shorter than 3.0 kb (*P* > 0.58).

### Comparing TL parameters across pregnancy outcomes

We measured SBmTL and TeSmTL in samples from the placenta and CAMs (CM and AM combined) donated by 36 women classified into four subgroups (n = 9 per each subgroup): (a) normal term pregnancies (gestational age 38.7 ± 0.9 weeks), (b) PTB (gestational age 33.6 ± 2.1 weeks), (c) PTBI (gestational age 29.8 ± 3.2 weeks), and (d) SGA pregnancies (gestational age 38.4 ± 1.19 weeks).

**Figure 6:**
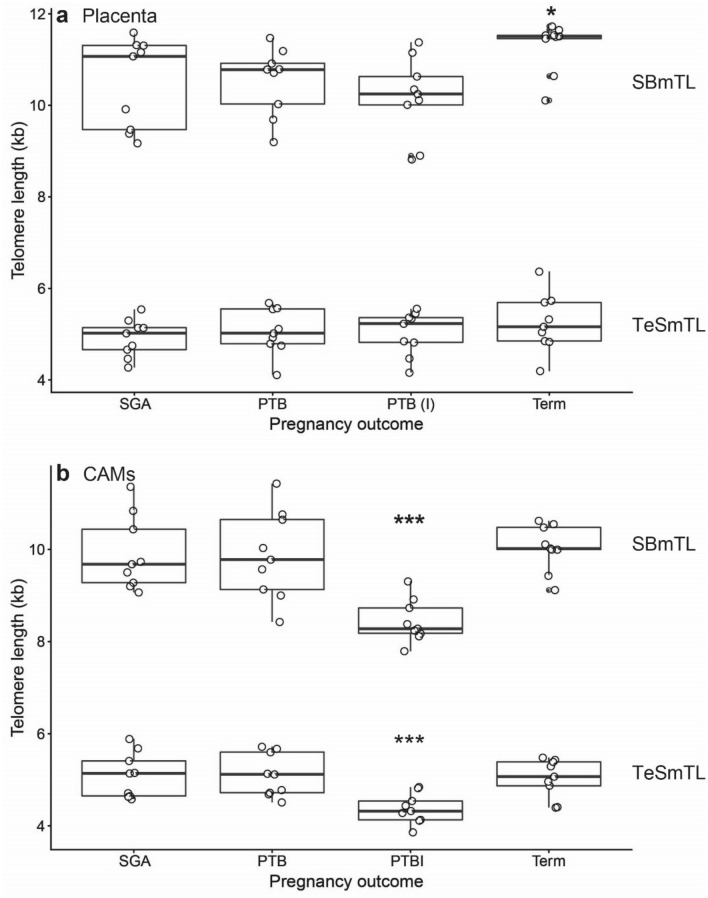
Mean telomere length by Southern blotting (SBmTL) and by the TeSLA (TeSmTL) for different pregnancy outcomes in the placenta (**a**) and the chorioamniotic membranes (CAMs) (**b**). Pregnancy outcomes (Groups): small for gestational age (SGA), pre-term birth (PTB), pre-term birth with intra-amniotic infection (PTBI). *P < 0.05; ***P < 0.001. n = 9 for each group.

SBmTL in placenta differed between the four groups (Fig. 6a; F_3,32_ = 3.16, *P* = 0.038). Placenta SBmTL was longer in the normal pregnancy group (least square mean ± SE: 11.3 ± 0.27 kb) than in the other three groups (PTB: 10.5 ± 0.27 kb; PTBI: 10.2 ± 0.27 kb; SGA pregnancy: 10.5 ± 0.27 kb). Post-hoc analysis revealed that only the difference in SBmTL between the PTBI group and the normal pregnancy group was significant (*t*_32_ = 2.96, *P* = 0.028), while there were no significant differences between the other groups (*P* > 0.16). In contrast to the results for SBmTL, TeSmTL did not differ significantly between the four pregnancy outcomes (F_3,32_ = 0.62, *P* = 0.61), although TeSmTL was also longer in the normal pregnancy group (least square mean ± SE: 5.24 ± 0.0.17 kb) than in the other three groups (PTB: 5.06 ± 0.17 kb; PTBI: 5.02 ± 0.17 kb; SGA pregnancy: 4.92 ± 0.17 kb). The proportion of telomeres shorter than 3.0 kb also did not differ between the pregnancy outcomes (F_3,32_ = 0.10, *P* = 0.96).

SBmTL in the CAMs differed between the four groups (Fig. 6b; F_3,32_ = 10.03, *P* < 0.0001). Post-hoc analysis showed SBmTL in CAMs to be significantly shorter in the PTBI group (least square mean ± SE: 8.44 ± 0.24) than in all other groups (*t*_32_ > 4.3, *P* < 0.001; Normal pregnancy: 10.04 ± 0.24 kb; PTB: 9.86 ± 0.24 kb; SGA pregnancy: 9.90 ± 0.24 kb). The same results emerged when comparing TeSmTL between pregnancy outcomes: there was significant variation between groups (F_3,32_ = 6.18, *P* < 0.002), and post-hoc showed TeSmTL in CAMs to be significantly shorter in the PTBI group (least square mean ± SE: 4.37 ± 0.14 kb) than in all other groups (*t*_32_ > 3.2, *P* < 0.02; Normal pregnancy: 5.03 ± 0.14 kb; PTB: 5.10 ± 0.14 kb; SGA pregnancy: 5.09 ± 0.14 kb). There was a trend for the same result when comparing the proportion of telomeres shorter than 3.0 kb between pregnancy outcomes (F_3,32_ = 2.39, *P* < 0.09) with the highest proportion of short telomeres in the PTBI group.

## Discussion

Our work shows that at the end of normal pregnancy, the human placenta displays evidence of TL-mediated replicative senescence. This conclusion is based on TeSLA measurements, coupled with TIF analyses. It is supported by the following set of observations: (a) diminished TeSmTL and increased proportion of telomeres shorter than 3 kb in the placenta between 18 weeks of gestation and full term; (b) a buildup in TIF between the two gestational stages; and (c) correlation between TeSmTL and TIF across the two gestational stages (18 weeks and full term). We could not reach this conclusion based on TL measurements by SB, as SBmTL did not show shortening between 18 weeks of gestation and full term. Moreover, at full term, SBmTL was longer in the placenta than in cord blood, confirming previous findings, which originally led to a conclusion that the placenta cannot be an aging organ^3,4^. However, SBmTL results do not include the critical population of telomeres shorter than 3 kb, which apparently signals TL-mediated replicative senescence^5,6^. The association of TIF with TeSmTL, but not with SBmTL, in the placenta at full term (and at 18 weeks of gestation) further supports this conclusion.

A recent study showed a buildup during gestation of short telomeres in the placenta and CAMs of mice^14^. Most mice have exceedingly long telomeres for their approximately 2-year lifespan; unless genetically engineered, mice are thus a sub-optimal model of TL-mediated replicative aging^15^. That said, the finding in mice support our observations in humans. We note that in and of itself, a buildup of short telomeres is insufficient to prove TL-mediated senescence. In our study, the CAMs displayed not only shortening of TeSmTL but also buildup of TIF between 18 weeks of gestation and full-term. However, we found no significant correlation between TeSmTL and TIF in the CAMs either at 18 weeks of gestation or at full term. We therefore cannot be certain, at present, that the human CAMs experience TL-mediated replicative aging during gestation.

TIF may result from processes other than TL shortening with repeated cell divisions^16^. As double stranded DNA breaks that occur in telomeres resist normal DNA repair activities, any genotoxic stress has the potential to activate a persistent DDR in telomeric sequences^7,17^. TL independent TIF formation can therefore occur due to a number of stresses, including those that that cause stalling of telomeric DNA replication forks. As repetitive and G-rich telomeric sequences are prone not only to developing a greater abundance of DNA lesions such as oxidative damage, but also form secondary structures called G4-DNA, telomeres are regions on chromosomes that are susceptible to replication fork stalling^18^. Failure to restart DNA replication at stalled forks often leads to formation of double stranded DNA breaks, which persist in telomeres and cause cellular senescence. In addition, increased levels or reactive oxygen species (ROS)^19^ or certain pro-inflammatory cytokines that raise intracellular ROS levels^20,21^ can also lead to TL independent TIF formation, likely due to mechanisms involving telomeric DNA replication stress. As a result, even a relatively long telomere can be damaged, resulting in TIF and senescence.

The mechanistic pathways that trigger term and preterm parturition and engender normal/abnormal intrauterine growth are still poorly understood. Based on mTL data^4,22–24^, it has been proposed that TL-mediated replicative aging of the placenta and CAMs might be a common pathway that contributes to the timing of term/preterm parturition and play a role in intra-uterine growth restriction^1–3^. Our pilot study examined, therefore, associations of TL parameters, measured by SB and TeSLA, with PTB, PTBI, and SGA pregnancies. We found that TL was short in the placenta (based on SBmTL) and CAMs (based on SBmTL and TeSmTL) only in PTBI. We cannot conclude, however, based on these findings that TL-mediated accelerated aging of the placenta, and CAMs in particular, contribute to PTBI. That is because the CAMs and placenta of PTBI display massive infiltration by maternal inflammatory cells^25^ with telomeres that are likely shorter than those in fetal structures. Therefore, in women with intra-amniotic infection, the inflammatory processes (e.g. inflammasome activation) induced by invading microbes may be the main cause of preterm labor and birth^26,27^.

Finally, we note the following study limitations: First, the sample size is small and for obvious reasons findings are based on cross-sectional data for evaluation of TL and TIF changes between 18 weeks of gestation and full term. Still, using TeSLA and TIF analysis, we were able to show that the placenta displays a buildup of short telomeres and telomere dysfunction at term. We were also able to show the same findings in CAMs but because of lack of correlations between TeSmTL and TIF in the CAMs, we are uncertain of the biological meaning of these findings in these tissues. Second, PTB and SGA are highly heterogeneous in nature and replicative aging might play a role in a subset of these obstetrical syndromes, which we might have missed because of the small sample size. Third, our findings are based on samples donated by subjects of African American and Hispanic ancestries. A recent study reported that placental mTL (measured by quantitative real-time polymerase chain reaction) was shorter in African Americans compared to whites of European ancestry^28^, while the consensus is that leukocyte mTL is longer in African Americans^29,30^. It is essential, therefore, to examine the profiles of SBmTL and TeSmTL in the placenta and CAMs across individuals from different ancestries.

In conclusion, TeSLA and TIF findings are consistent with the thesis that the human placenta experiences TL-mediated replicative aging during the course of pregnancy.

## Methods

### Subject characteristics

Samples were collected at the University Hospital (UH) of the New Jersey Medical School, Rutgers University, Newark New Jersey. At the UH, we collected samples from the placenta, CAMs, UCB, and maternal blood from 13 full-term normal newborns and their African American (n = 6) and Hispanic (n = 7) mothers [aged 27.62 ± 5.61 years (mean ± SD)]. We also obtained placenta and CAM samples from elective abortions donated by African American mothers (n = 10). In this set, the CM and the AM were collected as one tissue sample. The Rutgers University Institutional Review Board (IRB) approved research on the progression of pregnancy from 18 weeks of gestation to full term, which was performed at the New Jersey Medical School. All mothers for this component of this work signed a written informed consent.

Human placental and CAM samples for the pilot study of pregnancy outcomes were obtained at the Hutzel Women’s Hospital, Detroit Medical Center, Wayne State University (Step 3). The following groups of women (all African Americans, 9 women per group) were included: normal term; PTB; and PTBI based on high levels of IL-6 (> 2.6 ng/ml)^31^ and detectable microorganism in amniotic fluid^32–34^. SGA was defined as a term neonate born at 37–42 weeks of gestation with birthweight below the 10th centile for gestational age and with a cerebral placental ratio below the 5th centile for gestational age^35^. The collection and use of human materials for research purposes were approved by the IRB of Wayne State University. All participating women provided written informed consent prior to sample collection.

### Genomic DNA extraction

DNA was extracted using the Gentra Puregen DNA Extraction Kit (Qiagen, Valencia, CA, USA) and integrity was determined by resolving 20 ng of DNA on a 1% agarose. All samples passed the integrity test.

### Southern blotting of the terminal restriction fragments

Extracted DNA samples (3 μg each) was digested with restriction enzymes Hinf I and Rsa I (Roche Applied Sciences, Mannheim, Germany). Digested DNA samples and DNA ladders were resolved on 0.5% agarose gels for 16 h (2 V/cm). After electrophoresis, the mean length of the TRFs was measured by performing SB as previously described^11^.

### Telomere shortest length assay

TeSLA measurements were performed as previously described^12^. In brief, T4 DNA ligase (New England Biolabs, Ipswich, MA), 1 mM ATP, 10^−3^ μM of TeSLA-Ts and 50 ng of isolated genomic DNA were mixed in 1× CutSmart buffer (New England Biolabs, Ipswich, MA, USA) and incubated at 35 °C for 12–16 h. The mixture was then digested with CviAII, BfaI, NdeI, and MseI (New England Biolabs, Ipswich, MA, USA) to generate DNA fragments with 5′ AT and TA overhangs. Shrimp Alkaline Phosphatase (rSAP; New England Biolabs, Ipswich, MA, USA) was added to the digested mixture to remove 5′ phosphate from each DNA fragment. The mixture was combined with T4 DNA ligase, 1 mM ATP, 1 μM of AT adapter, and 1 μM of TA adapter in 1× CutSmart buffer to incubate at 16 °C for 12–16 h. Multiple PCR reactions were then performed using FailSafe Enzyme Mix (Lucigen, Middleton, WI, USA) with 1× FailSafe buffer H containing 0.25 μM AP/TeSLA-TP primers and 40 pg of ligated DNA. PCR products were resolved on a 0.85% agarose gel (1.5 V/cm for 19 h). After gel electrophoresis, SB is applied to detect amplified telomeres as previously described^36^. The algorithm of TeSLA detects each telomere band location, annotates the band size, and hence the raw data consist of a series of band sizes for each sample. The TeSLA software displays histogram of the telomere band size distribution and calculates relevant parameters.

### Immunostaining and telomere-immunoFISH

All procedures were performed essentially as described^8^. In brief, tissue samples were cryopreserved, cut, fixed in 4% paraformaldehyde/PBS, permeabilized in PBS/Tween (0.1%), and blocked overnight in 4% BSA/PBS. Samples were subsequently rinsed, dehydrated and nuclear DNA denatured in hybridization buffer containing Cy3 conjugated telomere specific peptide nucleic acid. Samples were then incubated with primary antibodies and subsequently with secondary antibodies as indicated. Slides were washed and mounted using DAPI containing mounting medium (Vector Laboratories, Burilngame, CA, USA). Immunostaining used primary polyclonal anti-53BP1 antibodies (Novus, Centennial, CO, USA) and secondary AlexaFluor 488-conjugated goat anti rabbit antibodies (Invitrogen, Carlsbad, CA, USA).

### Microscopy

Images were acquired using a Zeiss AxioObserver Z1, an AxioCam MRm camera (Zeiss, Pleasanton, CA, USA), and Zen Blue 2.3 software (Zeiss, Pleasanton, CA, USA). To analyze and quantitate co-localization between telomere signals and 53BP1 foci, images from 5 fields were acquired as z-series through the thickness of telomere signals (4–6 images, 0.5 μm optical slices) using a motorized stage and a 63X/1.4 oil immersion lens. To quantitate 53BP1 foci, images from 12 fields were acquired through the thickness of 53BP1 foci as z-stacks using a 40×/1.4 oil immersion lens. Stacks were merged into a single image using the Zen software for easier counting.

### Statistics

Data were analyzed using (mixed) general linear models using R Core Team^37^ and Tukey post-hoc tests. Proportions were arcsine square root transformed prior to analysis to ensure homoscedasticity.

### Ethics declarations

All subjects gave their informed consent for inclusion before they participated in the study.

### Approval for human experiments

This study received IRB approvals from Rutgers, New Jersey Medical School, The State University of New Jersey (Protocol No. 2018001630) and Wayne State University (Protocol No. 1011008995 and 0510003068). All participating women provided written informed consent prior to sample collection. All methods involving human participants were carried out in accordance with relevant guidelines and regulations.

## Supplementary Information

Supplementary Information

## Data Availability

The data that support the findings of this study are available from the corresponding author upon reasonable request.
