# Supplementary material for: Telomeres and replicative cellular aging of the human placenta and chorioamniotic membranes
Source: Sci Rep. 2021 Mar 4;11:5115. doi: 10.1038/s41598-021-84728-2 (PMC7933277; doi:10.1038/s41598-021-84728-2)
Supplement: Supplementary file 1 — Supplementary Information [file 41598_2021_84728_MOESM1_ESM.docx]

**Telomeres and Replicative Cellular Aging of the**

**Human Placenta and Chorioamniotic Membranes**

Tsung-Po Lai, Mark Simpson, Krunal Patel, Simon Verhulst, Jungsik Noh, Natalie Roche, Debra Heller, George Guirguis, Jerry W. Shay, Utz Herbig, Abraham Aviv

**SUPPLEMENTARY INFORMATION**

**Supplementary Figure S1-S5**

**Supplementary Table S1**

**Supplementary Table S2**

**
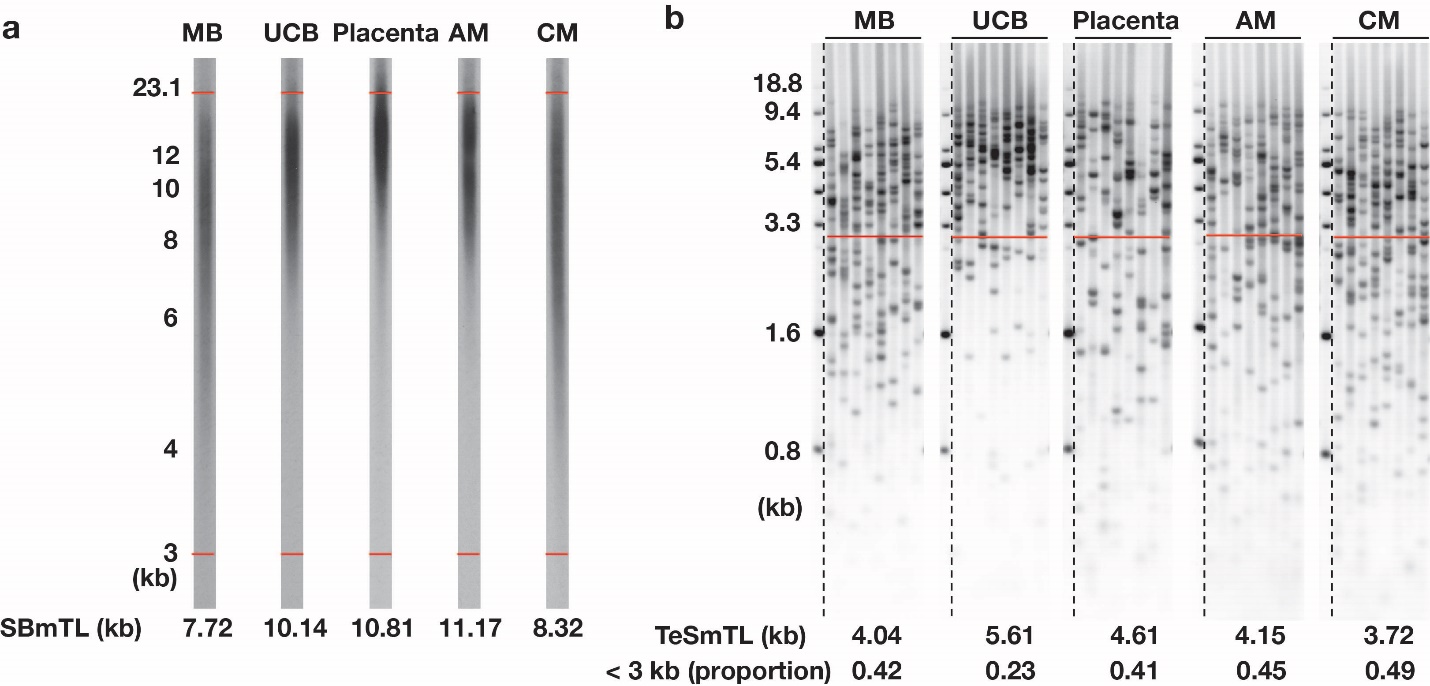
**

**Figure S1. Illustrations of Southern blotting (SB) and the Telomeres shortest length assay (TeSLA).** Sample are from the maternal blood (MB), umbilical cord blood (UCB), placenta, amniotic membrane (AM), and chorionic membrane (CM). SB and TeSLA measurements are from the same DNA sample from a mother and her newborn. For SB, each lane shows a single run for each sample. For TeSLA, each panel comprises seven lanes and the single telomeres are shown as bands. All lanes in each panel are derived from one DNA sample. In this way the TeSLA output generates sufficient bands required to ascertain reliability and reproducibility of the measurements. The red horizontal lines for SB display the scan limits between 20 and 3 kb. The red horizontal line for TeSLA show the 3 kb cutoff. Results mean TL by SB (SBmTL) (**a**), mean TL by TeSLA (TeSmTL) and proportion of telomeres shorter than 3 kb by TeSLA (**b**) are displayed at the bottoms of the lanes and panels. Molecular weight (MW) reference ladders are shown on left. The untrimmed images are presented in Fig. S5.

**
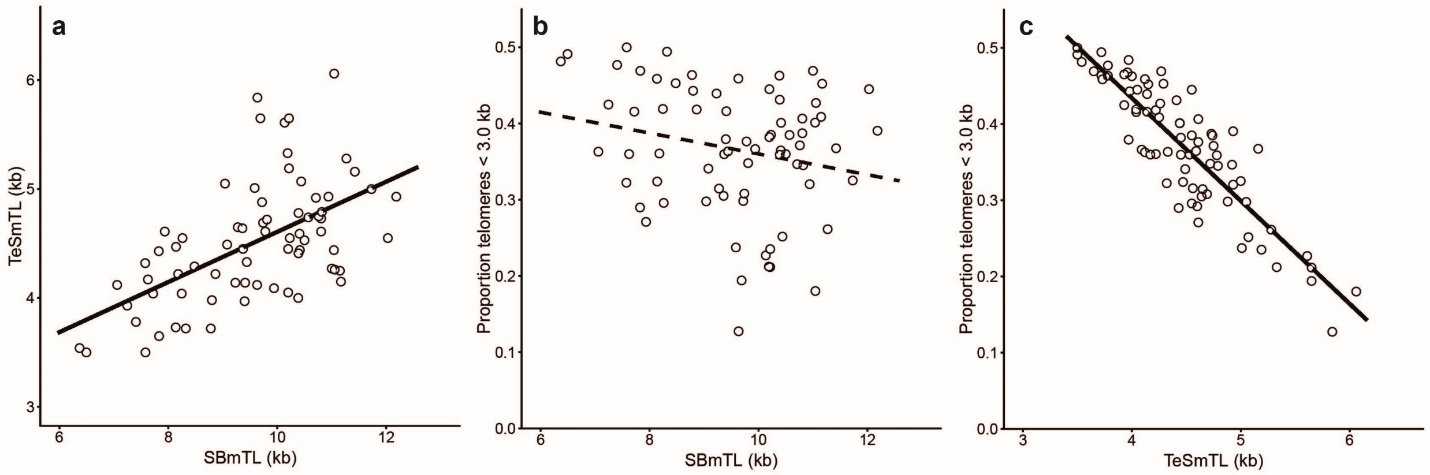
**

**Figure S2. Correlations between TL data generated by Southern blotting (SB) and the Telomeres Shortest Length Assay (TeSLA).** Panel **a** shows correlation (R^2^ = 0.32, P < 0.001) between mean TL by SB (SBmTL) and mean TL by TeSLA (TeSmTL). Panel **b** shows correlation (R^2^ = 0.05, P = 0.06) between SBmTL and proportion of telomeres shorter than 3 kb by TeSLA. Panel **c** shows correlation (R^2^ = 0.77, P < 0.001) between TeSmTL and proportion of telomeres shorter than 3 kb. n = 72.


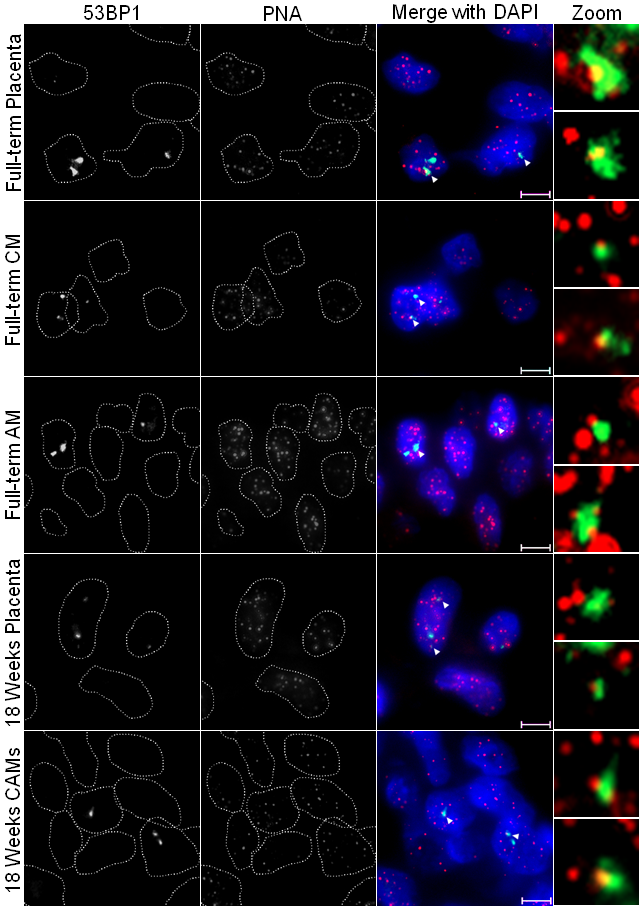


**Figure S3.** **Telomere dysfunction induced foci (TIF) in placenta and chorioamniotic membranes (CAMs).** Tissue sections from placenta, chorionic membrane (CM), amniotic membrane (AM) or jointly, CAMs were processed by immunoFISH to visualize 53BP1 foci (1st column, green) and telomeres (2nd column, red) with dashed lines to outline cell nuclei. Enlarged versions of indicated 53BP1-telomere co-localizations are shown to the right. Scale bars: 5 μm.


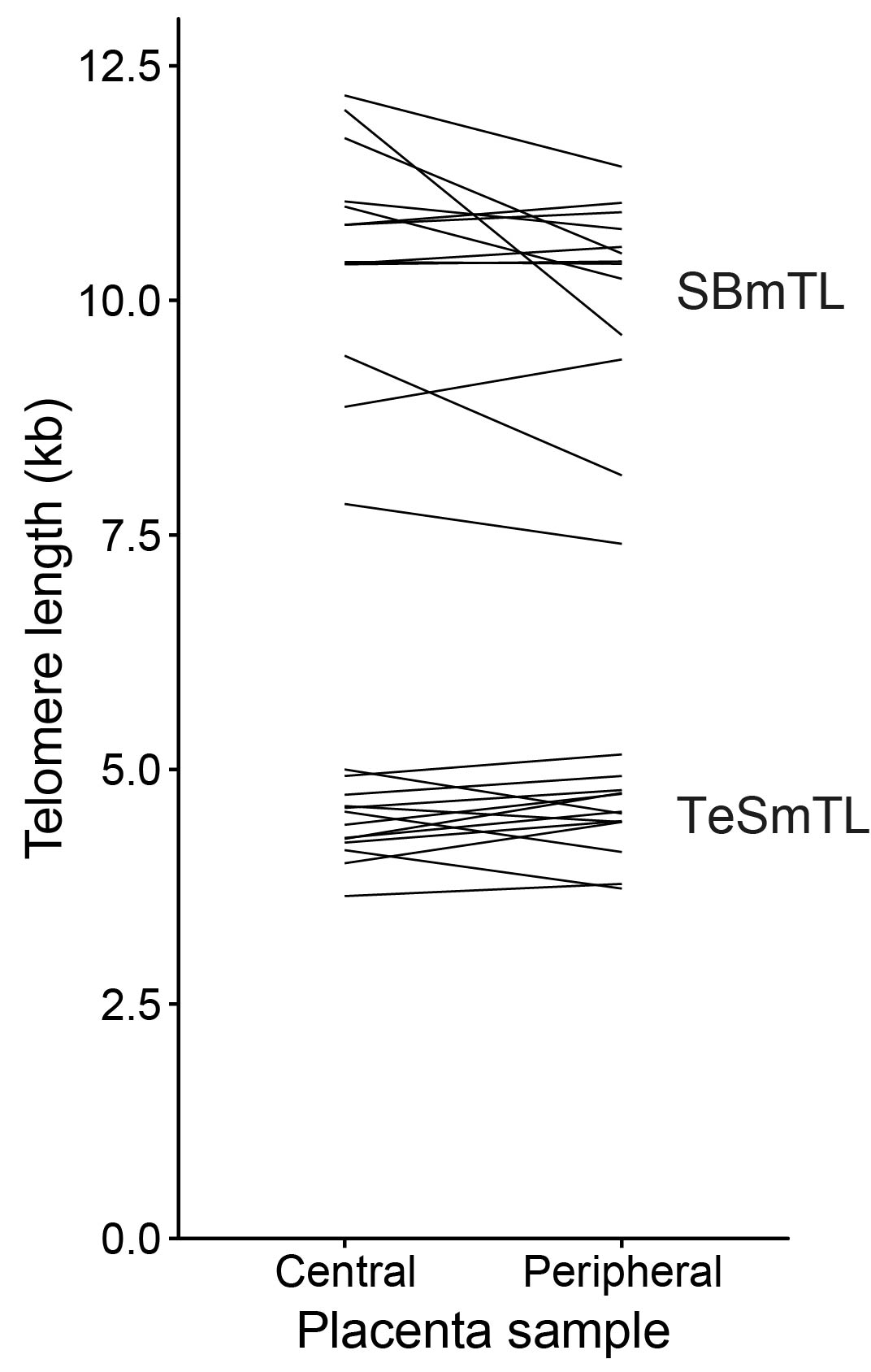


**Figure S4.** **Mean telomere length by Southern blotting (SBmTL), and by TeSLA (TeSmTL) in peripheral (PLP) versus central (PLC) placenta samples** (n = 12)**.** There was a trend for SBmTL to differ between PLP and PLC (P = 0.059), but no evidence for a difference in TeSmTL (P = 0.40).


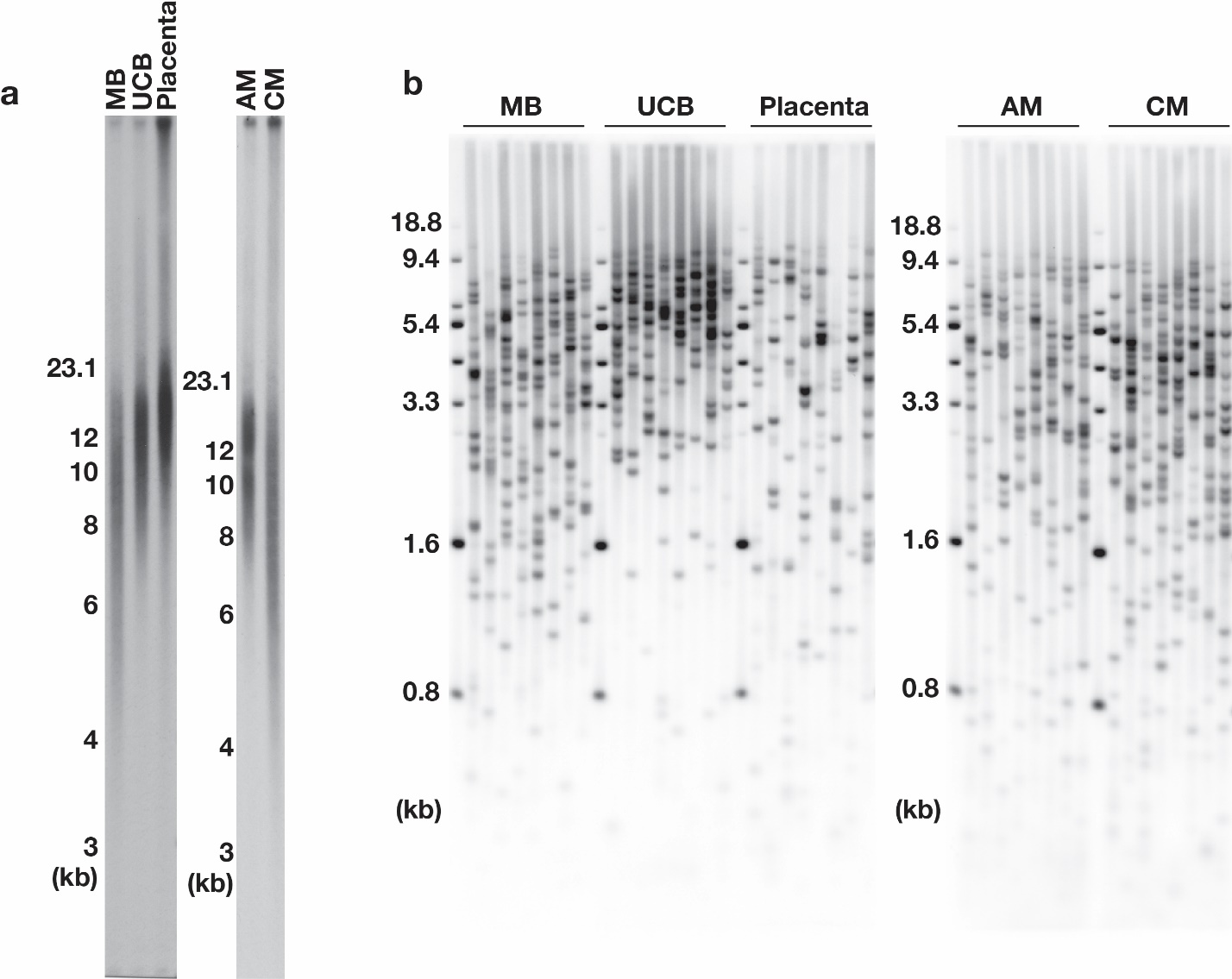


**Figure S5. The untrimmed image of (a) Southern blotting (SB) and (b) the Telomeres shortest length assay (TeSLA) shown in Fig. S1.** Molecular weight reference ladders are indicated.

**Table S1. Results of post-hoc tests comparing telomere length (kb) in different tissues at full-term, measured using A. Southern blotting (SBmTL) and B. TeSLA (TeSmTL).** Tissues: maternal blood (MB), umbilical cord blood (UCB), amniotic membrane (AM), chorionic membrane (CM), Placenta (PL).

**A. SBmTL**

**Contrast Estimate (SE) df *t* p**

MB - CB -1.948 (0.251) 42.0 -7.762 <.0001

MB - AM -2.638 (0.274) 42.8 -9.623 <.0001

MB - CM -1.410 (0.274) 42.8 -5.143 0.0001

MB - PL -2.848 (0.251) 42.0 -11.346 <.0001

CB - AM -0.689 (0.274) 42.8 -2.515 0.1062

CB - CM 0.539 (0.274) 42.8 1.965 0.3001

CB - PL -0.900 (0.251) 42.0 -3.584 0.0074

AM - CM 1.228 (0.286) 42.0 4.291 0.0009

AM - PL -0.210 (0.274) 42.8 -0.767 0.9386

CM - PL -1.438 (0.274) 42.8 -5.247 <.0001

**B. TeSmTL**

**Contrast Estimate (SE) df *t* p**

MB - CB -1.0838 (0.15) 48 -7.233 <.0001

MB - AM -0.3708 (0.15) 48 -2.474 0.1137

MB – CM -0.0308 (0.15) 48 -0.205 0.9996

MB – PL -0.2931 (0.15) 48 -1.956 0.3030

CB – AM 0.7131 (0.15)) 48 4.758 0.0002

CB – CM 1.0531 (0.15) 48 7.027 <.0001

CB – PL 0.7908 (0.15) 48 5.277 <.0001

AM – CM 0.3400 (0.15) 48 2.269 0.1729

AM – PL 0.0777 (0.15) 48 0.518 0.9851

CM – PL -0.2623 (0.15) 48 -1.750 0.4139

**Table S2: The list of abbreviations**

| Abbreviation | Explanation |
| --- | --- |
| AM | Amniotic membrane |
| CAM | Chorioamniotic membrane |
| CM | Chorionic membrane |
| DDR | DNA damage response |
| mTL | Mean telomere length |
| PNA | Peptide nucleic acid |
| PTB | Preterm birth |
| PTBI | Preterm birth with intra-amniotic inflammation/infection |
| ROS | Reactive oxygen species |
| SB | Southern blotting |
| SGA | Small-for-gestational age |
| TeSLA | Telomere shortest length assay |
| TIF | Telomere-dysfunction-induced DNA damage foci |
| TL | Telomere length |
| TRF | Terminal restriction fragment |
| UCB | Umbilical cord blood |
| 53BP1 | p53 binding protein 1 |
